# A Medical Causerie

**Published:** 1915-04-03

**Authors:** 


### A MEDICAL CAUSERIE.

The Scottish Medical Service Emergency Committee has issued some suggestions directed to the relief of the emergency created by the present shortage of medical practitioners. The first is that the public should co-operate by sending messages for the doctor as early in the day as possible, and by abstaining from urgent calls in circumstances where no actual emergency exists. This proposal is a somewhat ancient friend, but it has never secured a wide popular suffrage. A second appeal is to education authorities to release entirely or in part medical officers engaged in school inspection work so that they may be free to undertake ordinary practice. The committee, further, calls upon doctors who have retired from practice, but who are still "physically fit and willing," to send in their names and arrangements will be made to provide them with duties free from excessive strain. Last of all it is suggested that the younger members of the teaching staffs of medical schools should during the vacation give an opportunity to overworked practitioners to get a much needed holiday. All these proposals seem eminently practical and reasonable, and it may be hoped they will be widely adopted. There seems to be a general impression that the profession in Scotland is more efficiently organised than is the case south of the Tweed. This view was strongly presented at the time of the fight against the National Insurance Act, but the manner in which the opposition collapsed on that occasion hardly supported the claim.

The difficulties of the hospitals in reference to the supply of resident medical officers do not diminish. In more than one of the metropolitan hospitals the position is very acute, and there seems no option but to reduce activities as, for example, by closing the out-patient departments. It is even suggested that the visiting staff will have to arrange to divide the indoor work among its members who may be willing in turn to go into residence in the hospital premises. The numbers of women engaged as residents is increasing, but here also the supply is becoming exhausted. The Victoria Hospital at Dover has secured the help of Dr. Philippe Briffaux, of the University of Louvain, and in one of the London hospitals a young Japanese physician is in residence.

There are reasons to believe that some of the hospital staffs outside the official circle are taking steps to respond to the appeal of the Director-General for more doctors for the Army. Already these staffs are much depleted by the absence of men who have gone to the Front, but possibly the remaining members could help were work for them arranged in London. But the claim of the civil population must not be overlooked, for there can be no doubt that further diminution of hospital facilities in this direction would be an extremely serious matter. Between staffs already weakened by military claims and invasion of the wards by sick and wounded soldiers it is idle to pretend that the hospitals have been able fully to discharge their normal and ordinary work. The question of the payment of the hospitals for the reception and treatment of wounded soldiers still remains open, though it is understood that official negotiations are in progress. A related issue is the attitude the King's Fund is likely to take up in reference to non-occupied beds. Wards have at times been kept vacant so as to be at the service of the War Office when urgently needed. If such vacancies are to count against the hospitals when grants from the King's Fund come to be calculated, and if, at the same time, no payments are to be allowed by the War Office, the hospital finances may suffer from a double blow.

The protest of the City Coroner against the action of the Treasury in relation to "treasure-trove" is an interesting reminder of the duties of the Coroner outside his ordinary activities. It seems that during some excavations in the City a box containing a quantity of old jewellery was discovered, and that in some fashion or other the "powers that be" secured this and transferred it to the London Museum, where it is now on exhibition. In so doing, without any reference to the district Coroner, Dr. Waldo contends that his official responsibilities were unjustifiably invaded. Whether he will be able to make this protest good against the Treasury officials remains to be seen, but as a matter of doctrine there can be little doubt he is quite right. By a gradual change extending over some centuries the Coroner has come to be almost exclusively an officer engaged in inquiries concerning the cause of death in instances where this event has been more or less sudden and mysterious. In the popular judgment his function is to determine whether death has been due to natural or to non-natural causes, and in the latter circumstances he has to decide whether or not anyone has incurred criminal responsibility. But originally the Coroner was a Revenue officer engaged in the interests of the Crown, and the primary purpose of the inquest was to see whether or not the Crown could establish a claim against the property of the deceased. With a similar aim the Coroner kept his eye on stranded whales, royal sturgeons, shipwrecks, and "treasure-trove," ready to assert in each of these instances the rights of the Sovereign. All of these powers have now lapsed with the single exception of "treasure-trove," and the recent event suggests that this also is in danger. Dr. Waldo has just been elected President of the Coroners' Society, and therefore is in a good position to organise his colleagues to stand up for the rights of their craft. The struggle ought to be an interesting one, though it will scarcely engage much public sympathy. The late Mr. Troutbeck a few years ago conducted an inquiry on some "treasure-trove" discovered in Piccadilly, so that Dr. Waldo has a recent precedent for his encouragement.

